# Diseases of the Circulatory System

**Published:** 1905-04-22

**Authors:** 


### Progress in Medicine and Surgery.

### DISEASES OF THE CIRCULATORY SYSTEM.

M. H. Sicard[1] gives a description of 32 cases of ulcerative endocarditis, and reviews previous work. He recognises four types—first, the rapidly fatal cases with high temperature; secondly, those with irregular fever for weeks or months; thirdly, the cases where meningitis is dominant; and fourthly, those where endocardial symptoms appear with few constitutional ones. The micro-organisms found are varied, and the type of disease does not depend on the species present. Endocarditis generally exists before the attack commences, but primary cases do occur, and in a few there are no cardiac signs throughout the illness. The rapidly fatal cases show a leucocytosis generally over 15,000, and the slower ones have a lower one or a normal count. Treatment by serum gives very uncertain results. The disease is rare in young children, and differs from simple endocarditis in the extent to which the heart is attacked, in the frequency with which the right heart is involved, in the frequent presence of septic emboli, and of micro-organisms. However, it should be noted that in many definite cases micro-organisms have not been found.

**Pneumococcal Endocarditis.**—Preble[2] has made a study of this form of endocarditis. He finds that to it are due about one-fourth of all cases of bacterial endocarditis—that is, of all kinds except the endocarditis following arterio-sclerosis. This form, too, may be of any degree of severity, but in over three-fourths of the cases it is of the malignant type. The exudate is massive, but shows less tendency to ulceration than is seen in some other forms. It is peculiar, too, in attacking the aortic valves more often than the mitral, and in the frequency with which the right heart is involved as well as the left. Some sort of endocarditis occurs in one per cent. of all pneumonias, most frequently in young persons, and is generally due to the pneumococcus, but occasionally to other organisms. In this pneumococcal form meningitis is also present 60 times out of 100. The clinical picture otherwise is indistinguishable from that due to other organisms. It is favoured by the presence of old heart lesions, and may exist without the lungs being attacked by pneumonia. In many instances, however, when the two occur together, the pyrexia due to endocarditis commences three to six days after the pyrexia due to pneumonia has passed away. In some cases, indeed, there is no fever, and physical signs may often be absent. The endocarditis may continue for a few days or even for months. The pulse is usually rapid, but bradycardia is common. Endocarditis should be suspected where an irregular temperature follows a pneumonia without any cause. Blood cultures carefully repeated will at last show pneumococci. Recovery is probably more frequent than reported cases would show.

**Pericarditis.**—H. E. Lewis[3] points out that pericarditis should always be searched for in acute rheumatism, pneumonia, pleurisy, and Bright's disease. The friction sound may be made to disappear if the patient holds a full breath while pressure is made with the stethoscope, and this fact may be useful for distinguishing it from an endocardial sound. In very young children the affection may take the plastic form without any stage of effusion, and the writer believes that the majority of cases in children under five are tuberculous. In the treatment of pericarditis, counter-irritation, absolute rest, and small doses of Epsom salts frequently repeated in saturated solution are indicated. The prone position may be helpful. Paracentesis in extreme cases should be carried out. He describes a curious case in which a burn on the chest was followed by pericarditis and pleurisy. He performed paracentesis, and the patient made a good recovery. D. Riesman,[4] in a paper on the diagnosis of pericarditis, notices the difficulty of distinguishing some friction sounds from a double aortic murmur or from a presystolic mitral, and that it is hard to say why in some cases there should be severe pain and tenderness and none in others. A friction sound does not exclude the presence of fluid, and an apex-beat is sometimes perceptible though there is a large effusion. He considers that the point for aspiration differs in many cases. We may even puncture in the sixth space in the nipple line if the apex-beat can be felt above that point, or we may choose one of the right interspaces. A point where friction or heart sounds are very clear should be avoided, and the needle should be inserted slowly and arrested when the heart is felt to rub against its point. Pericarditis may simulate pneumonia, and here the compressed lung may give a patch of bronchial breathing. In other cases the pain may be felt in the abdomen and peritonitis diagnosed. Conversely in a few cases of peritonitis a friction sound may be heard over the pericardium. Acute delirium or mania is sometimes seen, and may be the first symptom. The pleural cavity may contain pus for some time before a general constitutional affection arises, but this follows very quickly upon similar pericardial trouble. Adherent pericardium is frequently undiagnosed. It may be suspected where friction sound has been heard over a large area and has disappeared, or in children where a chronic valve lesion and much hypertrophy exist, or in adults where ascites and œdema occur with a vigorously acting heart, or in quiet ascites where no cirrhosis or tuberculous trouble can be found. Among the signs are systolic retraction of the intercostal spaces and of the lower portion of the chest, marked undulation of the heart beat, and ascites before œdema of the legs commences.

**Valvular Disease.**—A. Morison[5] advises heroic doses of digitalis and strophanthus when we are dealing with a sound cardiac muscle which is stretched or enfeebled. He records remarkable successes in their use. The abdominal and thoracic muscles aid the action of the heart, and therefore after the removal of the fluid in ascites, strapping round the abdomen will do much in preventing its recurrence and will also increase the force of respiration, emptying the lungs and leading to more vigorous systole of the heart. The other voluntary muscles can be also enlisted in aid of the circulation by the Zander or Nauheim systems. It is, indeed, only in minor degrees of failure, or after convalescence, that these methods are useful. If the heart needs digitalis, baths and exercises are counter-indicated. With reference to the use of digitalis in aortic lesions, he would regard a small regular pulse with a hypertrophied heart and a systolic murmur as unlikely to benefit by it, but in aortic regurgitation if compensation fails there need be no hesitation in following Balfour's advice and using it. In extreme mitral stenosis and in valve troubles of the right heart digitalis should be replaced by strychnine, belladonna, or the nitrites. Especially is it harmful in chronic non-congenital disease of the right side. For adherent pericardium he hopes in the future that we may be able to call in surgical aid. P. A. Nightingale[6] reports a curious case of double aortic stenosis in a girl aged 17. The valves were thickened, and above them was a mass of vegetations and below them a thickened calcareous ring, causing a constriction such that the lumen was only 6 mm. across. The condition was probably of slow development and long standing. Slight dyspnœa had occurred two years before death, and became severe 21 months later. There was no evidence of acute rheumatism and only strepto- and staphylococci were found on the valves. The condition was probably due to a slow degenerative change following endocarditis. The organisms would be only secondary and acquired towards the end of life, though the recent vegetations were due to their action.

**Aneurysm.**—C. H. Melland[7] reports an instance of rupture of an aortic aneurysm, after which the patient lived for three weeks. Though such an occurrence is usually fatal at once, a few patients have lived months or years after a rupture of the sac and extensive hæmorrhage. Ferry has recorded nine cases, his own patient surviving for two years. In many instances small leakages occur, and in several cases life has been continued for a few hours or a couple of days after severe bleeding. In 1847 Lister recorded a death five months after a free hæmorrhage, and the trachea was found to be perforated in several places and plugged by clot; while Gairdner had a patient who lived five years after two severe bleedings. Melland's patient was a labourer aged 56 who had suffered from pain in the chest for five years when at work, and then brought up a very large amount of blood during some exertion. He was taken into the hospital in a collapsed state, recovered after a saline injection, but died three weeks later after repeated hæmorrhages. A saccular aneurysm was found on the convexity of the arch just beyond the left carotid which had extended between the trachea and the œsophagus. It had ruptured into the former with a vertical slit, three-eighths of an inch long. H. A. Ballance[8] describes the treatment of an aneurysm of the first part of the arch by the introduction of 8 feet of coiled silver wire into it and the passage of an electrolytic current through the aneurysm. The current was passed for 45 minutes and varied from 5 to 10 volts and 24 to 42 milliamperes, a large negative electrode being placed over the thigh. After four weeks' time the pain and the size of the tumour diminished greatly, and for a time the patient remained free from pain and able to get about. He died ten months after the operation. The aneurysm was found to be filled up with solid clot. It is probable that an unnecessary length of wire was used. Not only were coils found in the aneurysm embedded in clot, but the wire extended also both into the aorta and into the left ventricle, where it apparently caused little irritation and no clotting, though clotting took place freely in the sac.

[1] Amer. Jour. Med. Sci., Nov. [2] Ibid. [3] Med. Rec., Sept. 24. [4] Amer. Jour. Med. Sci., Sept. [5] Edin. Med. Jour., Oct.–Nov. [6] Lancet, Nov. 19. [7] Ibid. [8] Ibid., Oct. 1.

